# Nintedanib Inhibits Endothelial Mesenchymal Transition in Bleomycin-Induced Pulmonary Fibrosis via Focal Adhesion Kinase Activity Reduction

**DOI:** 10.3390/ijms23158193

**Published:** 2022-07-25

**Authors:** Wen-Kuang Yu, Wei-Chih Chen, Vincent Yi-Fong Su, Hsiao-Chin Shen, Huai-Hsuan Wu, Hao Chen, Kuang-Yao Yang

**Affiliations:** 1 Department of Chest Medicine, Taipei Veterans General Hospital, Taipei 11217, Taiwan; wkyu2@yahoo.com.tw (W.-K.Y.); wcchen2@vghtpe.gov.tw (W.-C.C.); hcshen3@vghtpe.gov.tw (H.-C.S.); purplewings0401@gmail.com (H.-H.W.); asura811218@gmail.com (H.C.); 2 School of Medicine, College of Medicine, National Yang Ming Chiao Tung University, Taipei 11217, Taiwan; bsbipoke@hotmail.com; 3 Institute of Emergency and Critical Care Medicine, College of Medicine, National Yang Ming Chiao Tung University, Taipei 11217, Taiwan; 4 Department of Internal Medicine, Taipei City Hospital, Taipei 11217, Taiwan; 5 Cancer Progression Research Center, National Yang Ming Chiao Tung University, Taipei 11217, Taiwan

**Keywords:** bleomycin-induced pulmonary fibrosis, endothelial mesenchymal transition, focal adhesion kinase, idiopathic pulmonary fibrosis, nintedanib

## Abstract

Idiopathic pulmonary fibrosis (IPF) is a progressive interstitial lung disease (ILD). Pulmonary fibroblasts play an important role in the development of IPF. Emerging evidence indicates that pulmonary endothelial cells could be the source of pulmonary fibroblasts through endothelial mesenchymal transition (EndoMT), which contributes to pulmonary fibrosis. EndoMT is a complex process in which endothelial cells lose their expression of endothelial markers and give rise to the characteristics of mesenchymal cells, including morphological fibroblast-like change and the expression of mesenchymal markers, which result in cardiac, renal, and dermal fibroses. Furthermore, EndoMT inhibition attenuates pulmonary fibrosis. Herein, we demonstrate that nintedanib, a tyrosine kinase receptor inhibitor, ameliorated murine bleomycin (BLM)-induced pulmonary fibrosis and suppressed the in vivo and in vitro models of EndoMT. We demonstrated that the activity of focal adhesion kinase (FAK), a key EndoMT regulator, increased in murine lung tissues and human pulmonary microvascular endothelial cells after BLM stimulation. Nintedanib treatment inhibited BLM-induced FAK activation and thus suppressed both in vivo and in vitro BLM-induced EndoMT. Importantly, we found that the VEGF/FAK signaling pathway was involved in nintedanib regulating EndoMT. These novel findings help us understand the mechanism and signaling pathway of EndoMT to further develop more efficacious drugs for IPF treatment.

## 1. Introduction

Idiopathic pulmonary fibrosis (IPF) is a chronic, progressive, and devastating interstitial lung disease (ILD) with an incidence about 30–100 per 100,000 person-years. Although its etiology is not completely understood, multiple risk factors, including smoking, aging, infection, inflammation, and environmental exposure, are closely associated with IPF, because they result in progressive fibrosing ILD, the loss of lung function, respiratory failure, and eventually death [1,2,3,4]. The pathologic hallmarks of IPF comprise various dense fibrosis, fibroblastic foci with myofibroblast accumulation, and honeycomb change within the lung parenchyma [5,6]. Fibroblasts and myofibroblasts play an important role in IPF development, including the release of proinflammatory cytokines and the production of the extracellular matrix [7,8]. However, the origins of fibroblasts and myofibroblasts are controversial for their heterogenous phenotypes [9,10]. Therefore, investigating the mechanisms of fibroblast/myofibroblast differentiation, migration, accumulation, and activation in the lung parenchyma is of great importance to ameliorate IPF severity.

Emerging evidence indicates that endothelial cells could be the source of fibroblasts through endothelial mesenchymal transition (EndoMT) [11,12,13,14,15]. EndoMT is a complex process in which endothelial cells lose their expression of endothelial markers (e.g., platelet endothelial cell adhesion molecule-1 (PECAM-1/CD31) and vascular endothelial cadherin (VE-cadherin)), and give rise to the characteristics of mesenchymal cells, including morphologic change and protein expression (e.g., α-smooth muscle actin (α-SMA) and vimentin) [11,12,13,14,15]. EndoMT inhibition attenuates pulmonary fibrosis [14,16,17,18], but the mechanisms are less well understood.

Nintedanib is a triple tyrosine kinase receptor inhibitor that targets platelet-derived growth factor receptor (PDGFR), fibroblast growth factor receptor (FGFR), and vascular endothelial growth factor receptor (VEGFR), and has been approved for the treatment of IPF and systemic sclerosis-associated ILD [19,20,21,22]. In a rat experimental pulmonary artery hypertension study, nintedanib suppressed the progression of vascular remodeling through EndoMT inhibition and therefore reduced the disease severity [23]. However, whether the inhibitory effect of nintedanib on EndoMT moderates pulmonary fibrosis has not been investigated.

Our overarching hypothesis was that nintedanib inhibits EndoMT and contributes to the amelioration of pulmonary fibrosis. We present that the expression of endothelial markers remarkably decreased with a concomitant increase in the expression of mesenchymal markers in the murine bleomycin (BLM)-induced pulmonary fibrosis model and during the BLM-induced EndoMT of human pulmonary microvascular endothelial cells (HPMECs) in vitro. Nintedanib treatment diminished pulmonary inflammation/fibrosis and inhibited EndoMT. Moreover, we identified that the degree of focal adhesion kinase (FAK) phosphorylation was upregulated in homogenized lung tissues from BLM-treated mice and in BLM-treated HPMECs, whereas nintedanib treatment downregulated FAK phosphorylation. Taken together, these findings demonstrated that nintedanib inhibits EndoMT by regulating FAK activity and attenuates BLM-induced pulmonary fibrosis.

## 2. Results

### 2.1. Nintedanib Alleviated BLM-Induced Pulmonary Injury and Fibrosis

**Figure 1:**
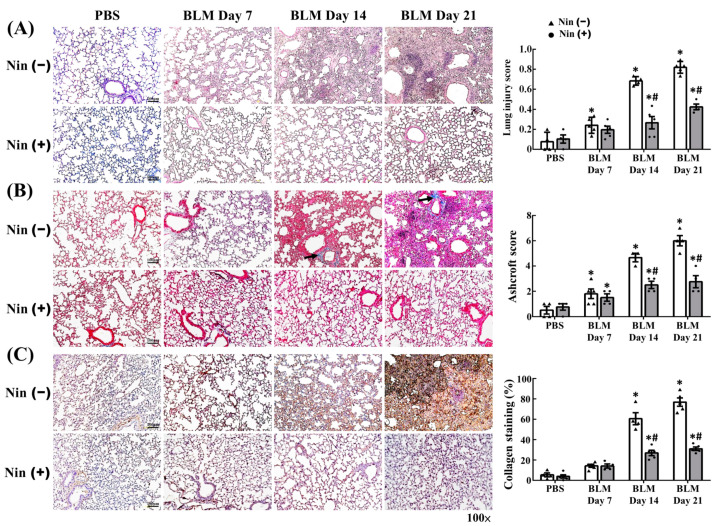
Nintedanib ameliorates BLM-induced pulmonary injury and fibrosis. (**A**) Representative hematoxylin and eosin staining images and lung injury scores of BLM-treated murine lung sections with or without nintedanib treatment. (**B**) Representative Masson’s trichrome staining images and Aschroft scores of BLM-treated murine lung sections with or without nintedanib treatment. Black arrows indicate collagen. (**C**) Representative IHC staining images and semi-quantification of collagen-1 in BLM-treated murine lung sections with or without nintedanib treatment. BLM, bleomycin; IHC, immunohistochemistry; Nin, nintedanib; PBS, phosphate-buffered saline. Scale bars, 100 µm. *n* = 3–5 in each group. Data are presented as mean ± standard deviation. * *p* < 0.05 vs. PBS, and # *p* < 0.05 vs. BLM (two-tailed Student’s *t*-test).

Mice were treated with BLM via intratracheal instillation (i.t.) to investigate its effect on pulmonary injury and fibrosis. Hematoxylin and eosin (H&E) staining of lung tissues showed alveolar septal congestion and extensive inflammatory cell accumulation in the alveolar space after BLM treatment (Figure 1A). The immunohistochemistry (IHC) staining of myeloperoxidase (MPO) in lung tissues clearly revealed neutrophil accumulation on day 7, 14, and 21 in mice treated with BLM compared with mice without BLM treatment. (Figure S1). Lung injury scores were also substantially higher on days 14 and 21 in mice treated with BLM compared with mice without BLM treatment (Figure 1A). Masson’s trichrome staining of lung tissues revealed that BLM treatment resulted in marked collagen accumulation (Figure 1B). Quantitative histological analysis for pulmonary fibrosis showed that the Ashcroft scores were statistically higher on days 7, 14, and 21 in mice treated with BLM compared with mice without BLM treatment (Figure 1B). Similarly, IHC staining showed that collagen-1 expression in murine lung tissues increased on days 14 and 21 in mice treated with BLM compared with mice without BLM treatment (Figure 1C). Afterward, the effect of nintedanib on pulmonary injury and fibrosis was explored. The BLM-treated mice were fed with 50 mg/kg nintedanib once daily for 5 days per week for 3 weeks [24]. The nintedanib-treated BLM-induced mice clearly showed lesser pulmonary injury in H&E-stained lung tissues and remarkably lower lung injury scores on days 14 and 21 compared with BLM-treated mice without nintedanib treatment (Figure 1A). The immunohistochemistry (IHC) staining of myeloperoxidase (MPO) in lung tissues clearly revealed reduced neutrophil accumulation on day 7 and 14 in BLM-stimulated mice treated with nintedanib compared with only BLM-stimulated mice. (Figure S1). Our previous study also reported that the levels of IL-1β and macrophage inflammatory protein-2 (MIP-2) increased in lung tissues after BLM stimulation. Nintedanib treatment reverted these changes induced by BLM [24]. Furthermore, the Masson’s trichrome staining of lung tissues showed that nintedanib treatment reduced the collagen accumulation and the Ashcroft scores on days 14 and 21 after BLM treatment compared with the BLM-treated mice without nintedanib treatment (Figure 1B).

### 2.2. Nintedanib Regulated the Expression of Mesenchymal and Endothelial Markers in BLM-Treated Mice

**Figure 2:**
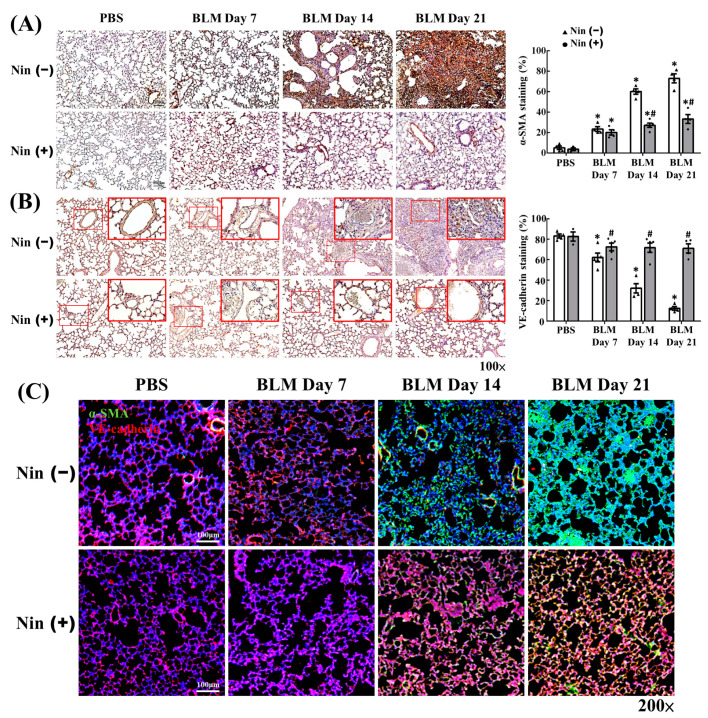
Nintedanib regulates the expression of vascular and mesenchymal markers in murine lungs treated with BLM. (**A**) Representative IHC staining images and the semi-quantification of α-SMA in BLM-treated murine lung sections with or without nintedanib treatment. (**B**) Representative IHC staining images and semi-quantification of VE-cadherin in BLM-treated murine lung sections with or without nintedanib treatment. Insert shows 4-fold magnification of indicated area. Scale bars, 100 µm. *n* = 3–5 in each group. Data are presented as mean ± standard deviation. * *p* < 0.05 vs. PBS, and # *p* < 0.05 vs. BLM (two-tailed Student’s *t*-test). (**C**) Representative IF staining images of VE-cadherin and α-SMA in BLM-treated murine lung sections with or without nintedanib treatment. Scale bars, 100 µm. Green, α-SMA; red, VE-cadherin; blue, DAPI. α-SMA, alpha smooth muscle actin; BLM, bleomycin; IF, immunofluorescence; IHC, immunohistochemistry; Nin, nintedanib; PBS, phosphate-buffered saline; VE-cadherin, vascular endothelial cadherin.

**Figure 3:**
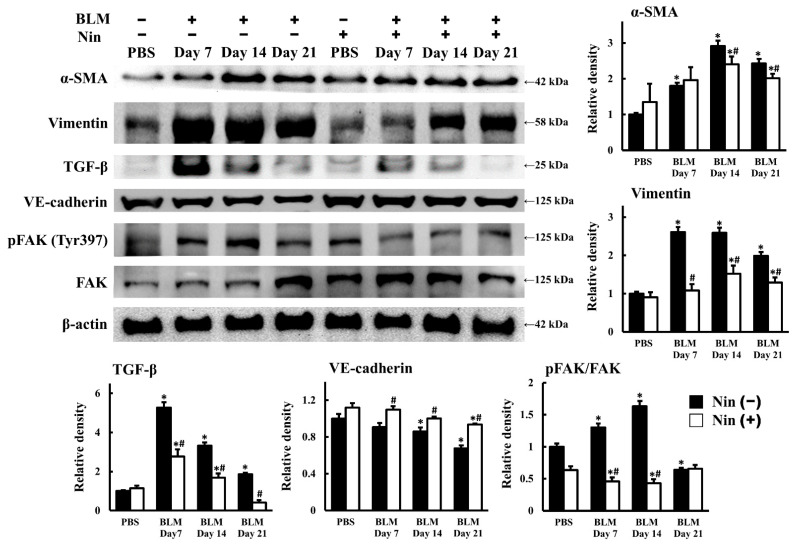
Nintedanib reverses BLM-induced changes in protein expression in murine lungs. Western blot and semi-quantitative analysis of protein expression in BLM-treated murine lung homogenates with or without nintedanib treatment. Western blot was performed using the indicated antibodies. *n* = 3–5 in each group. α-SMA, alpha smooth muscle actin; BLM, bleomycin; FAK, focal adhesion kinase; Nin, nintedanib; pFAK, phosphorylated focal adhesion kinase; TGF-β, transforming growth factor beta; VE-cadherin, vascular endothelial cadherin. Data are presented as mean ± standard error of mean. * *p* < 0.05 vs. PBS, and # *p* < 0.05 vs. BLM (two-tailed Student’s *t*-test).

BLM-induced pulmonary fibrosis could be attenuated by EndoMT inhibition [14,16,17,18]. Thus, we wanted to clarify whether nintedanib could suppress EndoMT in mice treated with BLM. The IHC staining of murine lung tissues showed that the expression of α-SMA, a mesenchymal marker, increased and the expression of VE-cadherin, an endothelial marker, decreased after BLM treatment (Figure 2A,B). Nintedanib treatment remarkably inhibited the α-SMA upregulation and VE-cadherin downregulation induced by BLM treatment (Figure 2A,B). The immunofluorescence (IF) staining of murine lung tissues also demonstrated that the intensity of α-SMA expression increased and that of VE-cadherin expression decreased after BLM treatment, but these changes were reverted by nintedanib treatment (Figure 2C). The levels of protein expression in murine lung homogenates were analyzed by Western blot, and the results are shown in Figure 3. The protein levels of surrogate mesenchymal markers, including α-SMA and vimentin, increased, whereas that of the endothelial maker, VE-cadherin, decreased after BLM treatment. Nintedanib treatment inverted the changes induced by BLM (Figure 3). Collectively, these findings indicate that nintedanib regulates the expression of endothelial and mesenchymal markers in BLM-treated murine lung.

### 2.3. Nintedanib Reduced FAK Activity in Murine Lung after BLM Intratracheal Instillation

Our data showed that nintedanib inhibited BLM-induced pulmonary fibrosis. Therefore, we sought to investigate the mechanism and signaling pathway in greater depth. The protein level of transforming growth factor-β (TGF-β), a fibrosis regulator, was upregulated in lung homogenates after BLM treatment, whereas nintedanib treatment downregulated the protein level of TGF-β (Figure 3). The IHC staining of murine lung tissues showed that the expression of active Smad2 (phospho-Smad2, pSmad2), a direct mediator of TGF-β signaling, increased after BLM treatment (Figure S2). Nintedanib treatment remarkably inhibited the activation of Smad2 induced by BLM treatment (Figure S2). FAK activity is increased in the lung tissues of patients with IPF [25,26]. FAK is a 125-kDa non-receptor tyrosine kinase that regulates cell signaling for adhesion, migration, proliferation, and survival [27,28,29]. In the present study, the expression of active FAK (phospho-FAK, pFAK) in the lung tissues also increased in the mice treated with BLM compared with the mice without BLM treatment. Moreover, the inhibition of FAK phosphorylation suppresses BLM-induced pulmonary fibrosis [30,31]. Our results are in accordance with previous reports, that is, the expression of pFAK in murine lung homogenates increased on days 7 and 14 after BLM treatment compared with the mice without BLM treatment. Furthermore, FAK phosphorylation was inhibited after nintedanib treatment (Figure 3). Taken together, these finding indicated that nintedanib might ameliorate BLM-induced pulmonary fibrosis through the reduction of FAK phosphorylation.

### 2.4. Nintedanib Alleviated BLM-Induced In Vitro EndoMT

**Figure 4:**
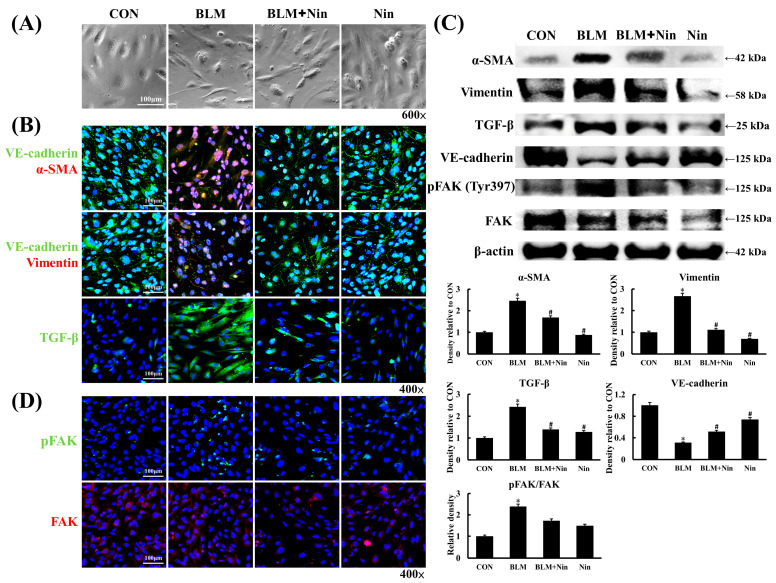
Nintedanib inhibits in vitro BLM-induced EndoMT. (**A**) Representative phase-contrast images of HPMECs treated with BLM or nintedanib alone or in combination. (**B**) Representative IF staining images of α-SMA, VE-cadherin, vimentin, and TGF-β in HPMECs treated with BLM or nintedanib alone or in combination. Scale bars, 100 µm. Green, VE-cadherin and TGF-β; red, α-SMA and vimentin; blue, DAPI. (**C**) Western blot and semi-quantitative analysis of protein expression in HPMECs treated with BLM or nintedanib alone or in combination. Western blot was performed using the indicated antibodies. *n* = 3–5 in each group. Data are presented as mean ± standard error of mean. * *p* < 0.05 vs. control, and # *p* < 0.05 vs. BLM (two-tailed Student’s *t*-test). (**D**) Representative IF staining images of FAK and pFAK in HPMECs treated with BLM or nintedanib alone or in combination. Scale bars, 100 µm. Green, pFAK; red, FAK; blue, DAPI. α-SMA, alpha smooth muscle actin; BLM, bleomycin; CON, control; EndoMT, endothelial mesenchymal transition; FAK, focal adhesion kinase; HPMEC, human pulmonary microvascular endothelial cell; IF, immunofluorescence; IHC, immunohistochemistry; Nin, nintedanib; pFAK, phosphorylated focal adhesion kinase; TGF-β, transforming growth factor beta; VE-cadherin, vascular endothelial cadherin.

HPMECs were co-cultured with or without nintedanib for 60 min and then stimulated with BLM for 6 h to determine whether nintedanib inhibits BLM-induced in vitro EndoMT. The HPMECs transformed into an elongated, spindle-shaped appearance, which resembles fibroblast-like appearance, after BLM stimulation (Figure 4A). This transformation was inhibited in the nintedanib treatment group (Figure 4A). IF staining of HPMECs showed that the intensity of α-SMA and vimentin expression increased, whereas that of VE-cadherin expression decreased after BLM stimulation. These changes were inverted in the nintedanib treatment group (Figure 4B). The protein expression of HPMECs was analyzed by Western blot as shown in Figure 4C. In agreement with earlier results, the protein expression levels of α-SMA and vimentin increased and that of VE-cadherin decreased after BLM stimulation. Nintedanib treatment reversed the changes induced by BLM (Figure 4C). The IF staining of HPMECs showed that the intensity of TGF-β expression increased after BLM stimulation, and nintedanib treatment decreased the intensity of TGF-β expression induced by BLM (Figure 4B). The protein level of TGF-β was also upregulated after BLM stimulation, and nintedanib treatment downregulated the protein level of TGF-β (Figure 4C).

### 2.5. Nintedanib Mediated the VEGFR/FAK Axis to Regulate BLM-Induced In Vitro EndoMT

**Figure 5:**
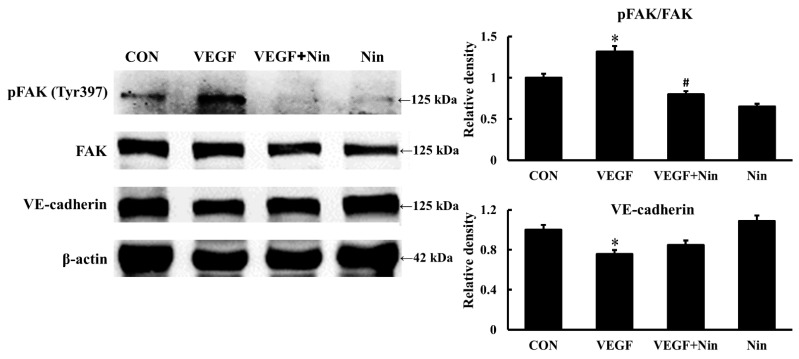
Nintedanib suppresses VEGF-induced FAK activation. Western blot and semi-quantitative analysis of protein expression in HPMECs treated with VEGF or nintedanib alone or in combination. Western blot was performed using the indicated antibodies. *n* = 3–5 in each group. CON, control; FAK, focal adhesion kinase; HPMEC, human pulmonary microvascular endothelial cell; Nin, nintedanib; pFAK, phosphorylated focal adhesion kinase; VE-cadherin, vascular endothelial cadherin; VEGF, vascular endothelial growth factor. Data are presented as mean ± standard error of mean. * *p* < 0.05 vs. control, and # *p* < 0.05 vs. VEGF (two-tailed Student’s *t*-test).

Our results showed that FAK phosphorylation increased in murine lung after BLM treatment. Yuan et al. reported FAK phosphorylation increased in an in vitro EndoMT experiment on human aortic endothelial cells; furthermore, the blockage of FAK phosphorylation inhibited EndoMT [32]. In the present study, HPMECs were co-cultured with or without nintedanib for 60 min and then stimulated with BLM for 6 h to explore whether nintedanib suppresses FAK phosphorylation and subsequently downregulates EndoMT in vitro. Western blot and the IF staining of HPMECs showed that the protein expression of pFAK after BLM stimulation was upregulated compared with the control, and nintedanib treatment downregulated the expression of pFAK (Figure 4C,D). Vascular endothelial growth factor (VEGF) mediates FAK phosphorylation and thus regulates endothelial cell functions, including cell adhesion and migration during angiogenesis and vascular permeability [33,34,35]. Therefore, we investigated whether nintedanib suppresses FAK phosphorylation through VEGFR inhibition. HPMECs were pretreated with nintedanib for 60 min and then stimulated with VEGF for 6 h. Western blot of HPMECs showed that FAK expression increased after VEGF stimulation but decreased after nintedanib treatment (Figure 5). Together, the results indicate that nintedanib could inhibit EndoMT by regulating the VEFGR/FAK axis.

### 2.6. Nintedanib Attenuated IPF Serum-Induced In Vitro EndoMT

**Figure 6:**
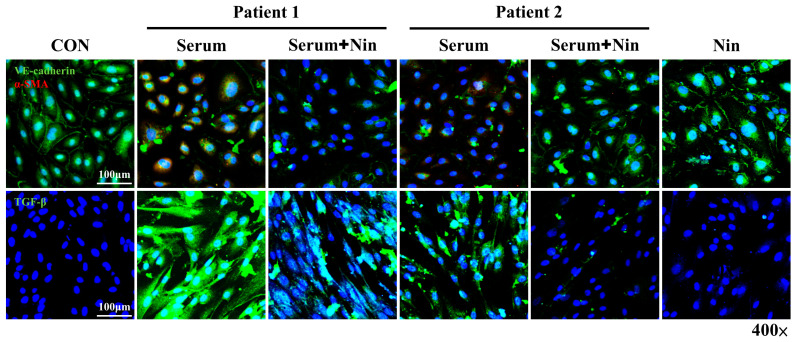
In vitro EndoMT is promoted by IPF but inhibited by nintedanib. Representative IF staining images of VE-cadherin, α-SMA, and TGF-β in HPMECs treated with IPF serum (from patients with IPF) or nintedanib alone or in combination. Scale bars, 100 µm. α-SMA, alpha smooth muscle actin; EndoMT, endothelial mesenchymal transition; HPMEC, human pulmonary microvascular endothelial cell; IPF, idiopathic pulmonary fibrosis; TGF-β, transforming growth factor beta; VE-cadherin, vascular endothelial cadherin. Green, VE-cadherin; red, α-SMA; blue, DAPI.

Serum from patients diagnosed with systemic sclerosis or sepsis could induce in vitro EndoMT [36,37]. Hence, we investigated whether IPF serum induces EndoMT or not. The IF staining of HPMECs after IPF serum stimulation is shown in Figure 6. The treatment of HPMECs with serum from patients with IPF resulted in the simultaneous downregulation of endothelial markers and the upregulation of mesenchymal markers (Figure 6). The intensity of TGF-β expression also increased after IPF serum stimulation (Figure 6). Importantly, nintedanib treatment reversed these changes. In summary, these findings indicate that nintedanib can inhibit IPF serum-induced in vitro EndoMT

## 3. Discussion

IPF is an irreversible and dismal disease in which normal pulmonary architecture is gradually lost and replaced by fibrotic tissue with fibroblast/myofibroblast proliferation and accumulation. Therefore, elucidating the mechanisms and mediators that contribute to fibrosis is of great importance to ameliorate and treat this disease. The transition of endothelial cells to fibroblasts through EndoMT occurs in the presence of injury or stimulation and results in organ fibrosis, including heart, kidney, and lung fibroses [11,12,13,15]. In addition, based on a murine experimental pulmonary fibrosis model, a substantial portion of pulmonary fibroblasts originate from pulmonary vascular endothelium [14]. In the present study, we showed the upregulation of α-SMA and vimentin and the downregulation of VE-cadherin in in vitro and in vivo BLM-induced pulmonary fibrosis. In addition, endothelial and mesenchymal markers (CD31 and α-SMA) were concurrently found in pulmonary arterial endothelium from patients with IPF [38]. The finding indicates the presence of EndoMT in experimental pulmonary fibrosis and IPF. Therefore, a therapeutic target against EndoMT may be a potential treatment for pulmonary fibrosis.

Nintedanib, an intracellular tyrosine kinase inhibitor, was licensed for the treatment of IPF and systemic sclerosis-associated ILD because of its clinical efficacy in reducing pulmonary function decline, decreasing acute exacerbation, and slowing disease progression [19,20,21]. In the in vitro and in vivo experimental pulmonary fibrosis models, nintedanib inhibited EndoMT-induced pulmonary fibroblast migration, proliferation, differentiation, and recruitment from alveolar epithelial cells [39,40,41,42,43]. Studies regarding the effects of nintedanib on the endothelium have focused on the inhibition of angiogenesis and increase in permeability [44,45,46]. Recently, nintedanib treatment reduced the disease severity in an experimental pulmonary arterial hypertension model through EndoMT inhibition [23]. In the present study, we extended this finding and found that nintedanib can reverse the downregulation of endothelial marker (VE-cadherin) and the upregulation of mesenchymal markers (α-SMA and vimentin) in BLM-treated murine lung. Moreover, we discovered that nintedanib can suppress in vitro BLM-induced EndoMT. These findings, together with the earlier report that a considerable portion of pulmonary fibroblasts originate from pulmonary endothelium in BLM-induced pulmonary fibrosis, implied that nintedanib ameliorates BLM-induced pulmonary fibrosis at least partially through EndoMT inhibition.

FAK is an integrin-associated cytoplasmic tyrosine kinase that plays an important role in cell adhesion, migration, proliferation, and angiogenesis [27,28,29,33,47]. FAK is also essential in the regulation of organ fibrosis, including heart and kidney fibroses [48,49,50,51]. pFAK is highly expressed in pulmonary myofibroblasts and pulmonary vascular endothelium from the lung biopsies of patients with IPF [30]. Furthermore, the administration of the inhibitor that blocked FAK phosphorylation attenuated murine pulmonary fibrosis induced by BLM [25]. In accordance with previous reports, our finding showed that pFAK expression increased in murine lungs after BLM treatment. We presented the novel finding that nintedanib treatment inhibits FAK phosphorylation and thus ameliorates BLM-induced pulmonary fibrosis. A previous study reported that FAK siRNA inhibits the high glucose-induced EndoMT of human aortic endothelial cells [32]. Similarly, we observed that pFAK was highly expressed in HPMECs after BLM stimulation and nintedanib treatment suppressed FAK phosphorylation. Together, these findings support the concept that nintedanib inhibits BLM-induced EndoMT by reducing FAK phosphorylation.

The associations of serum biomarkers with disease severity and prognosis, as well as the effects of serum biomarkers on diseases, have been studied for many years. A higher serum TGF-β level might induce the EndoMT of portal vein endothelium and is associated with portal vein stenosis in patients with idiopathic portal hypertension [52]. Serum leukotriene B4 (LTB4) level is also higher in patients with systemic sclerosis and induced EndoMT-related pulmonary and dermal fibroses [53]. In the current study, we also observed that serum from IPF patients induced the EndoMT of HPMECs and nintedanib administration reversed this change. Indeed, specific serum biomarkers, alone or in combination, can induce EndoMT, but the mechanism and signaling pathway require further study.

EndoMT is a complex process and encloses different mechanisms. In our study, we reported that nintedanib inhibited EndoMT through the reduction of FAK activity. However, other mechanisms, including protein-tyrosine phosphatase 4A3 (PTP4A3)/VEGF [54] and heat shock protein 90 (HSP90)/Akt signaling pathways [55,56] might be involved in the intricate EndoMT. Further experiments are necessary in the future to evaluate and compare with our study.

## 4. Materials and Methods

### 4.1. Experimental Animals

Male C57BL/6 mice, 6–8 weeks of age, were purchased from the National Experimental Animal Center (Taipei City, Taiwan) and maintained in standard plastic animal cages with husk bedding, a 12-h light/dark cycle, free access to food and water, specific pathogen-free conditions, and 25 ± 2 °C temperature.

### 4.2. Experimental Protocol of Murine BLM-Induced Pulmonary Fibrosis

The male C57BL/6 mice were placed individually in an induction chamber and were anesthetized with 4% isoflurane in 100% oxygen with a deliver rate of 0.6 LPM (liter per minute) until the loss of their righting reflex. Then the male C57BL/6 mice as BLM-induced pulmonary fibrosis murine model received one dose of 1.5 U/kg BLM sulfate (B5507, Sigma-Aldrich, St. Louis, MO, USA) in 50 μL of phosphate-buffered saline (PBS) by intratracheal instillation (i.t.) [56]. Mice in the PBS group only received 50 μL of PBS i.t. After 24 h, the mice were randomly grouped and treated with nintedanib (MedChemExpress, Monmouth Junction, NJ, USA) or 0.5% hydroxyethyl-cellulose (HEC). The nintedanib treatment group was given 50 mg/kg nintedanib (suspended in 0.5% HEC) via oral gavage once daily for 5 days per week for 3 weeks [24]. A number of 3 to 5 mice in each experimental group for a total of 8 groups were euthanized on day 1, 7, 14, and 21 for lung tissue collection and experiments according to the study design. All experiments and protocol were approved and conducted in accordance with the Institutional Animal Care and Use Committee (TVGH IACUC No. 2020-059; 8 January 2020).

### 4.3. Histology and Immunohistochemical (IHC) Staining

Lung sections were fixed in 4% paraformaldehyde for 10 min, embedded in paraffin, and cut into 4 μm-thick sections. Staining for VE-cadherin (1:100; ab33168, Abcam, Cambridge, UK), α-SMA (1:100; 14395-1-AP, Proteintech, Rosemont, IL, USA), collagen-1 (1:100; ab34710, Abcam, Cambridge, UK), MPO (1:50; sc-52707, Santa Cruz Biotechnology, Dallas, TX, USA), and pSmad2 (1:50; #18338, Cell Signaling Technology, Danvers, MA, USA) was performed using the Envision^®^ + Dual Link System-HRP (DAB+) kits (K4065, DAKO, Glostrup, DK). Briefly, the lung tissues were deparaffinized with xylene, dehydrated with ethanol, and heated in 0.01 M citrate buffer (pH 6.0). Endogenous peroxidase activity was inactivated in 3% H_2_O_2_ for 10 min at room temperature, and the sections were blocked with a blocking buffer. Secondary anti-rabbit antibody-coated polymer peroxidase complexes were subsequently applied for 30 min at room temperature, followed by substrate/chromogen treatment (K4065 kit) and further incubation for 5–15 s at room temperature. The sections were counterstained with hematoxylin (109249, Merck, Darmstadt, DE) for 10 s and washed in running water for 10 min. The sections were observed and photographed using an Olympus AX80 fluorescence microscope (Olympus, Melville, NY, USA). The percentage of IHC signal per photographed field was analyzed using the image processing software, Image-Pro Plus (Media Cybernetics, Inc., Rockville, MD, USA).

### 4.4. Lung Injury Score

Two investigators independently evaluated each H&E-stained slide in a blinded manner. Three-hundred alveoli were counted on each slide at 400× magnification to generate the lung injury score. Points within each field were assigned according to the predetermined criteria, and lung injury score was calculated as follows [57,58,59]: lung injury score = {(alveolar hemorrhage points/number of fields) + [2 × (alveolar infiltrate points/number of fields)] + [3 × (fibrin points/number of fields)] + (alveolar septal congestion/number of fields)}/total number of alveoli counted.

### 4.5. Masson’s Trichrome Staining

Lung tissues were fixed in 4% paraformaldehyde for 10 min, embedded in paraffin, and cut into 4 µm-thick sections. The Trichrome Stain Kit (ab150686, Abcam, Cambridge, UK) was used according to the manufacturer’s instructions.

### 4.6. Ashcroft Scale

The Ashcroft scale was used to quantify the severity of pulmonary fibrosis by histology. Points within each field were assigned according to the predetermined criteria [58,60].

### 4.7. Western Blot of Mouse Lung Homogenates

Mouse lung tissues were homogenized in lysis buffer with protease inhibitors (1862209, Thermo, Waltham, MA, USA). Equal amounts of protein homogenate were resolved on 7.5–10% sodium dodecyl sulfate–polyacrylamide electrophoresis gels and transferred onto polyvinylidene fluoride membranes. The blots were blocked in Tris-buffered saline with Tween (TBST) containing 5% milk and directly probed with the following primary antibodies: FAK (1:1000; #3285, Cell Signaling Technology, Danvers, MA, USA), pFAK (Tyr925; 1:500; #3284, Cell Signaling Technology, Danvers, MA, USA), TGF-β (1:500; #3711, Cell Signaling Technology, Danvers, MA, USA), VE-cadherin (1:1000; ab33168, Abcam, Cambridge, UK), vimentin (1:1000; V6630, Sigma-Aldrich, St. Louis, MO, USA), α-SMA (1:1500; 14395-1-AP, Proteintech, Rosemont, IL, USA), and β-actin (1:5000, 20536-1-AP, Proteintech, Rosemont, IL, USA). The blots were subsequently washed in TBST, incubated in horseradish peroxidase secondary antibodies and detected using an enhanced chemiluminescence substrate (Pierce Bio-chemicals, Thermo Fisher Scientific, Waltham, MA, USA). Each blot was exposed to a film, and the densitometry of immunoreactive bands was performed using ImageJ software (National Institute of Health, Bethesda, MD, USA).

### 4.8. Immunofluorescence (IF) Staining of Mouse Lung Tissues

Murine lung tissue sections were deparaffinized with xylene, dehydrated with ethanol, heated in 0.01 M citrate buffer (pH 6.0), and blocked with 3% fetal bovine serum (FBS; in PBS) for 60 min at room temperature. Then, VE-cadherin (1:1000; ab33168, Abcam, Cambridge, UK), α-SMA (1:1000; 14395-1-AP, Proteintech, Rosemont, IL, USA), and vimentin (1:1000; V6630, Sigma, Burlington, MA, USA) were applied overnight at 4 °C. On the next day, secondary antibodies, namely goat anti-rabbit IgG H&L (Alexa Fluor^®^ 488 (1:400; ab150077, Abcam, Cambridge, UK)) and Cy5^®^ (1:400; ab6564, Abcam, Cambridge, UK)), were incubated at 37 °C for 2 h. The slides were mounted on mounting medium with 4′,6-diamidino-2-phenylindole (DAPI; H-1200, Vector Laboratories, Burlingame, CA, USA) for nuclear staining. Cell images were captured on a Fluoview FV10i confocal microscope (Olympus, Japan).

### 4.9. In Vitro Culture and Stimulation of HPMECs

HPMECs were used as the in vitro BLM-induced EndoMT model. The cells were purchased from ScienCell Research Laboratories (#3000, Carlsbad, CA, USA); grown in endothelial cell medium (ScienCell Research Laboraties, Inc., Carlsbad, CA, USA), including endothelial cell growth supplement, penicillin/streptomycin solution, and 5% FBS; and incubated in room air with 5% CO2 and 95% humidity at 37 °C. The experiments were conducted on passage 2–3 HPMECs. HPMECs with a density of 5 × 10^4^ were seeded and cultured on each well of an 8-well chamber slide coated with fibronectin until confluency. Then, the HPMECs were pre-incubated with or without 400 mM nintedanib for 60 min and then stimulated with BLM (100 mU/mL) or VEGF (10 ng/mL; R&D, Minneapolis, MN, USA) for 6 h according to the experimental design. Cell cultures were removed for all liquid and washed twice with 1× PBS. The cells were fixed with 4% paraformaldehyde, permeabilized with 0.5% Triton X-100 (in PBS) and blocked with 3% FBS (in PBS) for 60 min at room temperature for IF staining.

### 4.10. IF Staining of HPMECs

Primary antibodies, namely, FAK (1:1000; #3285, Cell Signal, MA, USA), pFAK (1:1000; #3283, Cell Signal, MA, USA), VE-cadherin (1:1000; ab33168, Abcam, Cambridge, UK), α-SMA (1:1000; 14395-1-AP, Proteintech, Rosemont, IL, USA), and TGF-b (1:1000; ab92486, Abcam, Cambridge, UK), were applied overnight at 4 °C. On the next day, secondary antibodies, namely, goat anti-rabbit IgG H&L (Alexa Fluor^®^ 488 (1:400; ab150077, Abcam, Cambridge, UK) and Cy5^®^ (1:400; ab6564, Abcam, Cambridge, UK)), were incubated at 37 °C for 2 h. The culture slides were mounted using mounting medium with DAPI (H-1200, Vector Laboratories, Burlingame, CA) to obtain nuclear staining. Images of the cells were taken on a FluoView confocal microscope (FV10i, Olympus America, Melville, NY, USA).

### 4.11. In Vitro IPF Serum Treatment

HPMECs were used to test in vitro IPF serum treatment. HPMECs were cultured as previously mentioned until confluency. Then, the HPMECs were pre-treated with or without 400 mM nintedanib for 60 min and incubated with complete medium containing 20% IPF serum for 6 h. After the experiment, IF staining with indicated antibodies was performed as previously mentioned.

### 4.12. Statistical Analysis

The mice were prepared and studied concomitantly. Separate groups of mice (*n* = 3–5 per group) were used in IHC, Western blot, lung injury score, Ashcroft score, and IF analyses. Data are presented as mean ± standard error of mean for each experimental group. One-way ANOVA and Tukey–Kramer multiple comparisons test were used for the comparison of multiple groups, and Student’s t test was used for the comparison between two groups. *p* < 0.05 was considered significant.

## 5. Conclusions

In conclusion, our results demonstrated, for the first time, the involvement of FAK-dependent EndoMT in BLM-induced pulmonary fibrosis. Nintedanib treatment reduced FAK activation, suppressed the EndoMT process, and ameliorated BLM-induced pulmonary fibrosis. These novel findings help us understand the mechanism and signaling pathway of EndoMT in pulmonary fibrosis for the development of more efficacious drugs for IPF treatment.

## Data Availability

The datasets used and/or analyzed during the current study are available from the corresponding author upon reasonable request.

## Supplementary Materials

The supporting information can be downloaded at: https://www.mdpi.com/article/10.3390/ijms23158193/s1.
