# Supplementary figures and images for: Nintedanib Inhibits Endothelial Mesenchymal Transition in Bleomycin-Induced Pulmonary Fibrosis via Focal Adhesion Kinase Activity Reduction
Source: Int J Mol Sci. 2022 Jul 25;23(15):8193. doi: 10.3390/ijms23158193 (PMC9332002; doi:10.3390/ijms23158193)

# Figure S1

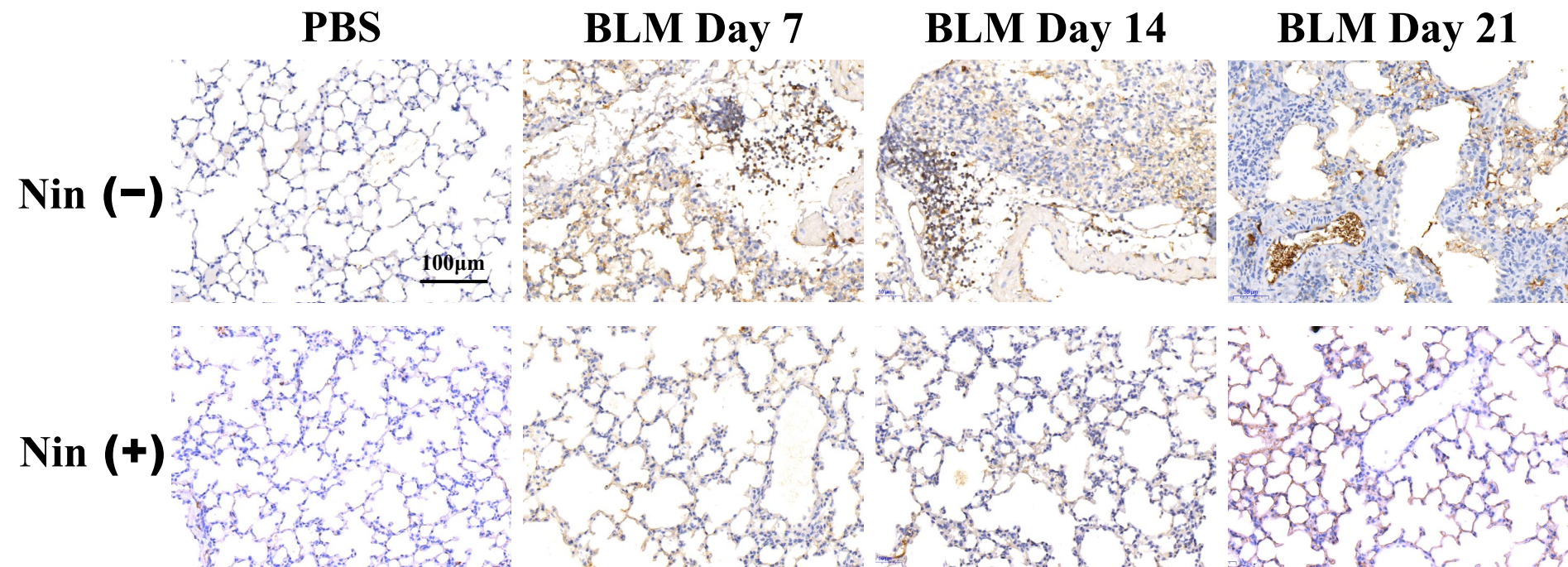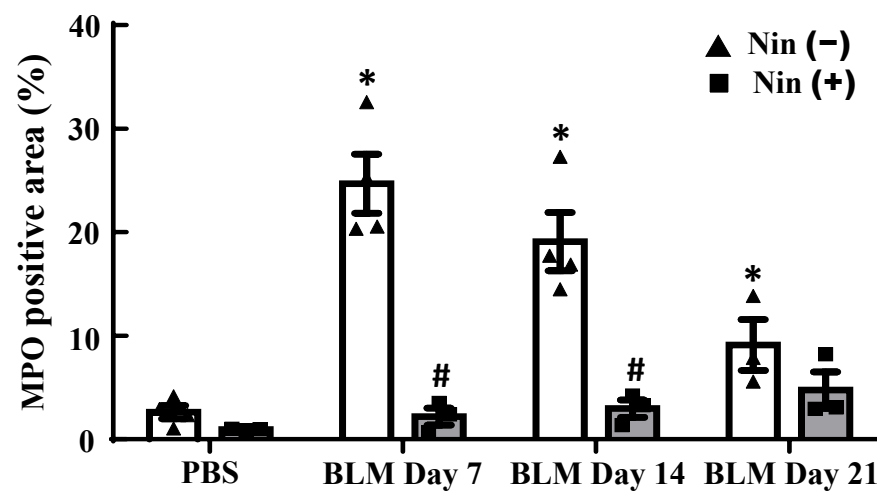

# Figure S2

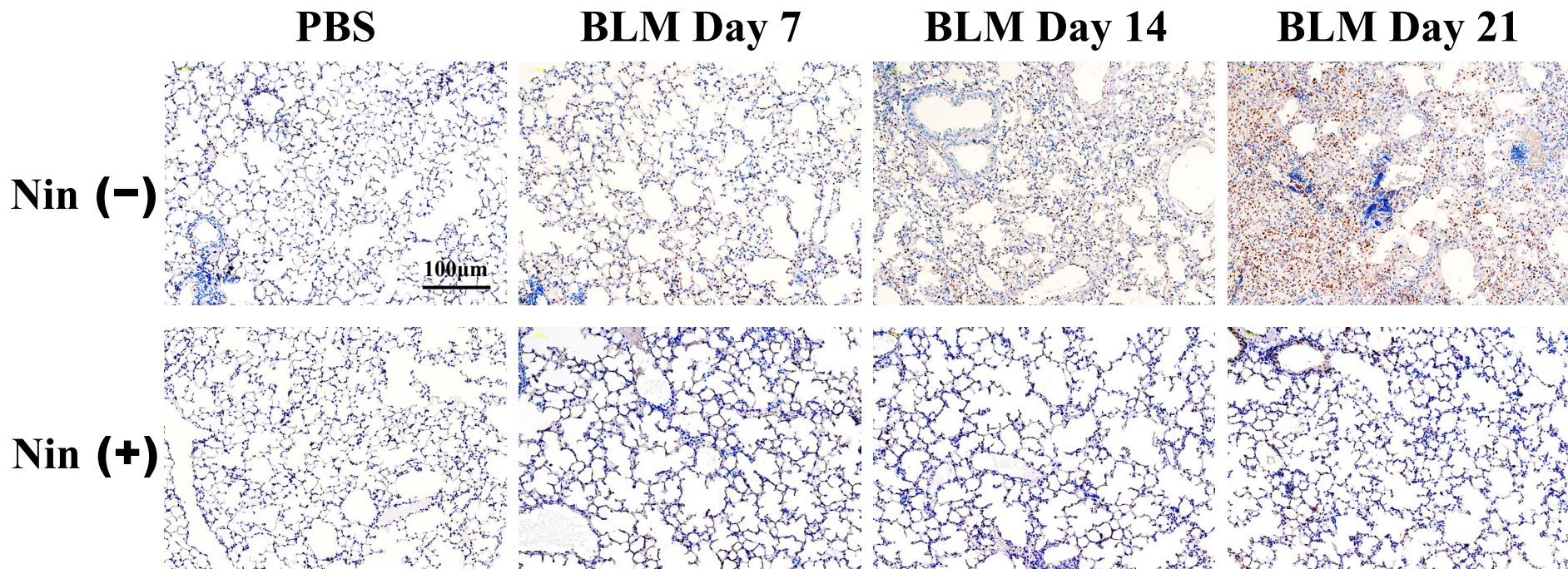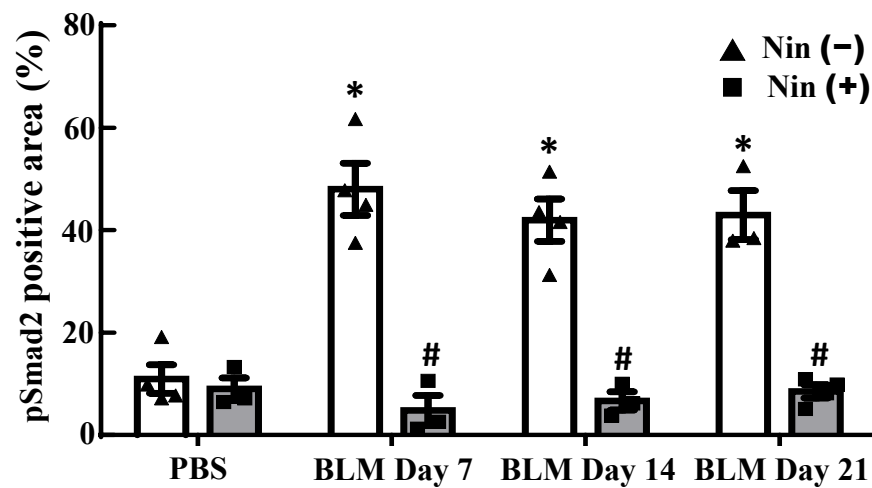

Supplement: Supplementary file 1 [file ijms-23-08193-s001.zip › ijms-1730145-supplementary.pdf]
